# Neoadjuvant chemoimmunotherapy as a potential therapeutic option in NSCLC UICC stage IIIA with multilevel N2 disease

**DOI:** 10.1016/j.rmcr.2022.101728

**Published:** 2022-08-14

**Authors:** Hruy Menghesha, Fabian Doerr, Georg Schlachtenberger, Andres Amorin Estremadoyro, Karin Töpelt, Thorsten Wahlers, Khosro Hekmat, Matthias Heldwein

**Affiliations:** a University of Cologne, Faculty of Medicine and University Hospital Cologne, Department of Cardiothoracic Surgery, Kerpener Strasse 62, 50937, Cologne, Germany; b University of Cologne, Faculty of Medicine, University Hospital Cologne, Department of Internal Medicine I, Center for Integrated Oncology Cologne/Bonn, Kerpener Strasse 62, 50937, Cologne, Germany

**Keywords:** Non-small cell lung cancer, Neoadjuvant, Immunotherapy, Surgical therapy

## Abstract

Lung Cancer is still one of the leading causes for cancer related death worldwide. The determination of an adequate therapeutic approach requests a precise staging, which contains computed tomography (CT) of the thorax, positron emission tomography computed tomography (PET-CT), cerebral magnetic resonance imaging (cMRI) and pulmonary function testing as well as the patient's opinion. In UICC stages I and II, if there is functional operability and technical resectability, the treatment of choice is primary surgery followed by adjuvant therapy depending on lymph node status, while patients in the metastatic stage IV, or with locally advanced, nonresectable disease are more likely to receive definitive chemoradiation therapy. The UICC Stage III (8th edition) combines a heterogeneous group of patients that remains the focus of discussion regarding the optimal therapeutic regimen, which ranges from primary surgical care to a neoadjuvant therapeutic approach, to definitive conservative treatment. Since March 2020, we have been treating a patient on an interdisciplinary basis who initially had a UICC stage IIIA multilevel N2 pulmonary adenocarcinoma and finally underwent successful surgery after a very good response to neoadjuvant chemoimmunotherapy. Our latest follow-up showed no evidence of recurrence. Similar to current ongoing studies our case shows, that neoadjuvant immunotherapy is a reasonable alternative to conventional neoadjuvant chemotherapy.

## 1 Introduction

Lung cancer is the most commonly diagnosed cancer and the leading cause of cancer death in female and male combined [1]. A careful histopathological staging is an important foundation in determining adjuvant therapy [2]. In the early UICC stages I and II there is a clear indication for primary surgical therapy followed by adjuvant chemotherapy or chemoradiation therapy [2]. In other cases, which show locally advanced growth or distant metastases, palliative systemic therapy is preferred [3]. In between, UICC stage III is a tumor stage that varies widely, particularly with regard to the distribution and extent of lymph node metastases, and its treatment is controversial [8]. The recommendation for neoadjuvant therapy, which until now has only included chemotherapy as the systemic component, is repeatedly made. The benefit and tolerability of immunotherapeutics in neoadjuvant therapy of lung cancer is increasingly being investigated and will be presented based on our case report.

## 2 Case report

**Fig. 1:**
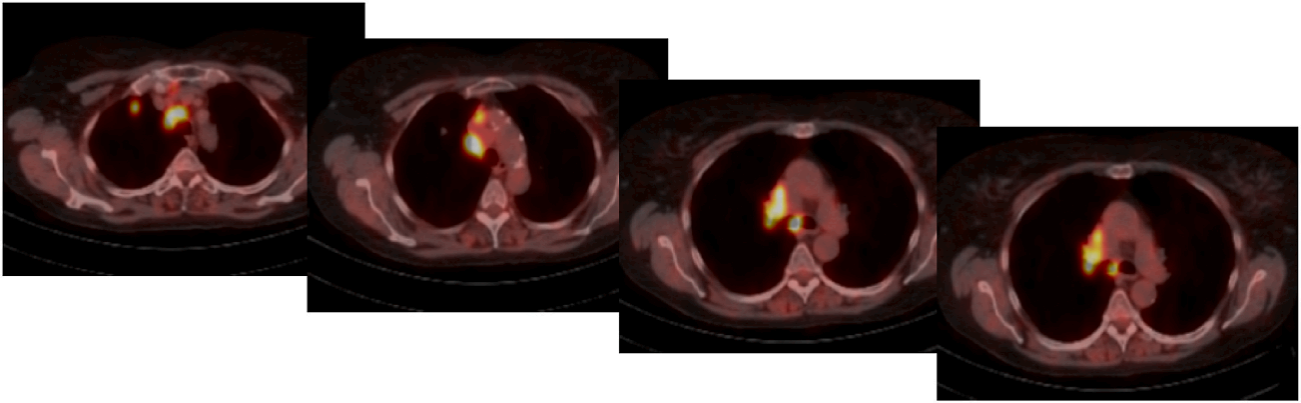
Initial PET-CT-Scan: Marked FDG-uptake of the primary lesion and mediastinal and hilar lymph nodes (N2, Robinson 3).

The presentation of a 61-year-old patient with a BMI of 27.4 kg/m^2^ (height: 171 cm; weight: 80 kg) was due to a diagnosed pulmonary adenocarcinoma of the right upper lobe. The diagnosis was confirmed by a transbronchial biopsy already in the pretreatment hospital. After EBUS sampling a pN2 situation was revealed (lymph-node station 4 right, lymph-node station 10 right). As secondary diagnoses, the patient presented with an abdominal aortic aneurysm of 55 mm with no progression in short term monitoring, rheumatoid arthritis, and migraine treated with medication. Further histopathologic, immunohistochemical, and molecular pathologic analysis revealed a pulmonary adenocarcinoma with a PD-L1 tumor proportion score of <1%, an EGFR and a BRAF wild type and TP53 mutation (p.H179R; p.E271). As part of guideline-recommended further staging, cerebral metastases were excluded by cMRI. However, the PET-CT performed before transbronchial biopsy and EBUS presented the diagnosed pN2 multilevel disease (Fig. 1).

**Fig. 2:**
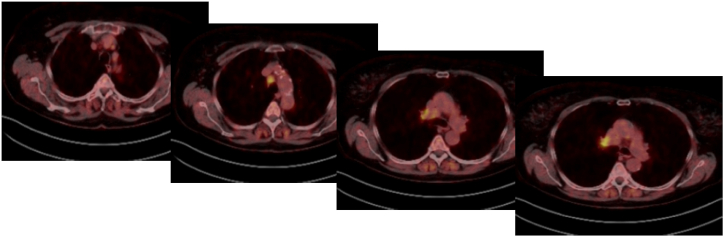
PET-CT-Scan after first two cycles of neoadjuvant chemoimmunotherapy: good response with reduced FDG-uptake.

After extensive discussion in MDT, the decision was made to use neoadjuvant chemoimmunotherapy with carboplatin, paclitaxel, and pembrolizumab in this UICC stage IIIA N2 patient, which was not guideline based. After two cycles a re-staging was conducted using PET-CT-Scan, which presented a good response to the established therapy with decreasing glucose metabolism in mediastinal and hilar lymph nodes and size reduction of the primary lesion (Fig. 2).

**Fig. 3:**
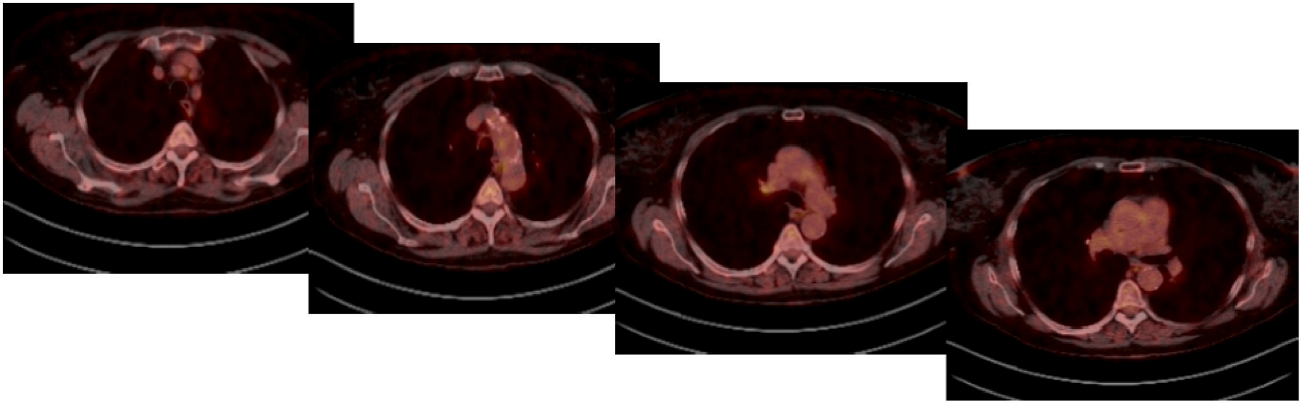
PET-CT-Scan after cycles 3 and 4 of neoadjuvant chemoimmunotherapy: No detectable FDG-uptake in lymph-nodes. Borderline elevated FDG-uptake in the primary lesion.

After renewed discussion about the further therapeutic procedure within the MDT, we initiated two more cycles of the described therapy. However, this had to be discontinued due to therapy-limiting side effects, which included autoimmune nephritis and hypothyroidism and were treated by systemic prednisolone application. Based on this, we performed restaging again, which showed a continued good response to the applied therapy and no distant metastasis (Fig. 3). Consequently, we decided not least due to the urgent request of the patient to perform surgical right upper lobe resection with systematic nodal dissection (SND) via a muscle-sparing anterior minithoracotomy 5 weeks after administration of the last dose of chemoimmunotherapy. Pulmonary function testing showed a borderline operability with a FEV1 of 2.05 l, a DLCO of 57% and a VO_2_max of 11.5 mL/kg/min.

Postoperatively, the patient was extubated in the operating room, and after a 24-h stay in the intensive care unit, she was transferred to the normal ward. The chest tube was removed on day 4 and the patient was discharged on day 6 after surgery.

Surgical therapy of abdominal aortic aneurysm was recommended after repeated angio-CT imaging at short interval by our colleagues in vascular surgery.

**Fig. 4:**
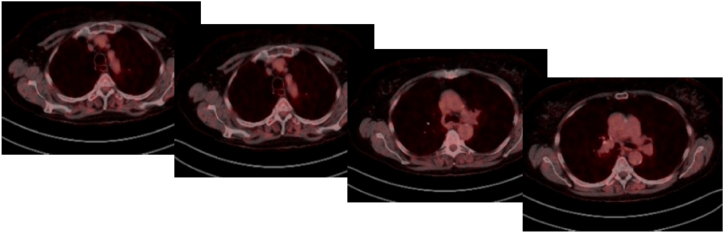
PET-CT-Scan with 1-year-follow up after resection of the right upper lobe: No detectable FDG avidity.

Histopathological analyses of the resected specimens showed a TNM stage of ypT0, ypN0 (0/15), L0, V0, Pn0, R0 and thus no detectable tumor activity (0% vital tumor cells). Finally, the patient could be included in the regular tumor follow-up, which still showed an inconspicuous 1-year follow-up (Fig. 4) to the last, which was confirmed by our last correspondence with the patient (27 months after diagnosis, 20 months after surgery).

## 3 Discussion

A sufficient oncologic therapy requires a detailed staging, which contains PET-CT-Scan, cMRI and ideally lymph-node biopsy [2]. If possible, the optimal solution is surgical resection, which may be supplemented by chemoradiation depending on the lymph-nodal status. UICC stage IIIA includes a very heterogeneous group of patients for whom an individualized therapeutic concept should be defined in the context of a MDT. In the case of N2 multilevel disease (Robinson 1–4) [4], discrimination should be made between the option of definitive chemoradiation, and neoadjuvant therapy followed by surgical resection. As in the case of our patient, the boundaries between the two therapeutic options and thus the optimal treatment are not always clear [5], so that a re-evaluation of the therapeutic concept in the course is useful. A patient who initially appears to be inoperable but who responds excellently to the applied therapy can possibly be ‘downstaged’ to an operable stage, as we were able to show. As we learned from the KEYNOTE-189 study, even patients with a PD-L1 status of <1% benefit from combination therapy with pembrolizumab in terms of overall survival and tumor response, which in the case of our patient led to the decision to adopt an individualized therapy approach [6]. In addition, the NADIM trial demonstrated that patients treated neoadjuvantly with nivolumab in addition to conventional chemotherapy had significantly better progression free survival [7]. At the same time, the Checkmate 816 trial demonstrated superiority over chemotherapy alone in terms of progression free survival and complete pathological response with comparable tolerability [8].

In the specific case of our patient, the sequence of surgical therapy for abdominal aortic aneurysm and NSCLC had to be discussed. This was done not least on the basis of the European guideline for the therapy of AAA, as well as a study by Brown et al. according to which the risk of rupture after 2 years is about 7.8%, whereas the risk of mortality of a patient with NSCLC at the stage of our patient is approximately 45% [9,10].

As ESMO Practice Guidelines recommend regarding pulmonary function testing, the VO_2_max of our patient was borderline with 11.5 mL/kg/min [2]. Nevertheless, the patient did not show any problem regarding the respiratory situation and an adequate exercise tolerance after discharge from inpatient treatment on postoperative day 6 and after 1-year follow-up.

## 4 Conclusion

As the case of our patient shows, especially the patient population of NSCLC patients in UICC stage IIIA is very heterogeneous and needs a detailed discussion in the MDT since there is no clear treatment guideline. The decision to use neoadjuvant chemoimmunotherapy may give patients in UICC stage IIIA N2, who would otherwise receive definitive conservative therapy, another treatment option. Only the surgical resection, which can be performed without elevated risk after neoadjuvant chemoimmunotherapy, can lead to a complete tumor freedom and is therefore a more meaningful component in our view.

## Funding

We acknowledge support for the Article Processing Charge from the DFG (German Research Foundation, 491454339).

## Declaration of competing interest

None.
